# In veteran outpatients, antibiotics remain significant risk factor for community-acquired *Clostridiodes difficile* infection

**DOI:** 10.1017/ash.2022.104

**Published:** 2022-05-16

**Authors:** Ukwen Akpoji, Brigid Wilson, Tayoot Chengsupanimit, Sunah Song, Taissa Bej, Robin Jump, Federico Perez

## Abstract

**Background:** An estimated 30% of antibiotic prescriptions in outpatient settings may be inappropriate. Antibiotic exposure increases an individual’s risk of *Clostridioides difficile* infection (CDI). To assess the prevalence of community-acquired CDI (CA-CDI) among patients without recent hospitalization and to examine the influence of outpatient antibiotic exposure on the risk of acquiring CA-CDI in this population, we examined a 2-year cohort of patients seen in primary care clinics at VA community-based outpatient clinics (CBOCs) associated with a large VA medical center. **Methods:** All primary care visits and nonvisit antibiotic prescriptions were identified in calendar years 2018–2019 as encounters of interest. Encounters occurring **Results:** We identified 84,787 patients with visits meeting our criteria. In this cohort, 3,533 patients were prescribed antibiotics at their encounter of whom 5 (0.14%) developed CA-CDI. Among the 81,254 patients who were not prescribed antibiotics, 15 (0.02%) developed CA-CDI, yielding an unadjusted CA-CDI odds ratio of 7.68 (95% CI, 2.50–19.82). p **Conclusions:** Although CA-CDI episodes were infrequent among VA outpatients with a CBOC visit in 2018–2019, the odds of CA-CDI were 7-fold greater in outpatients with antibiotic exposure than outpatients without antibiotic exposure. Antibiotic stewardship interventions that emphasize adverse events as a result of care provided in the outpatient setting, rather than as events limited to acute-care settings, may mitigate CDI risk.

**Funding:** This work was supported by the Merck Investigator Studies Program (MISP 59266 to F.P. and R.J.), and funds and facilities were provided by the Cleveland Geriatric Research Education and Clinical Center (GRECC) at the VA Northeast Ohio Healthcare System. The funders had no role in the design and conduct of the study; collection, management, analysis, and interpretation of the data; preparation, review, or approval of the manuscript; and decision to submit the manuscript for publication.

**Disclosures:**. All authors report no conflicts of interest relevant to this article. R.J. has received research funding from Pfizer; she has also participated in advisory boards for Pfizer and Merck.

